# PK/PD Modeling to Assess Rifaximin Clinical Dosage in a Mouse Model of *Staphylococcus aureus-*Induced Mastitis

**DOI:** 10.3389/fvets.2021.651369

**Published:** 2021-06-14

**Authors:** Honglei Wang, Chen Chen, Xiaojie Chen, Jingju Zhang, Yiming Liu, Xiubo Li

**Affiliations:** National Feed Drug Reference Laboratories, Feed Research Institute, Chinese Academy of Agricultural Sciences, Beijing, China

**Keywords:** *Staphylococcus aureus*, mastitis, rifaximin, PK/PD model, Monte Carlo simulation

## Abstract

*Staphylococcus aureus* (*S. aureus*) is a common pathogen that causes mastitis, an infection of the milk-secreting tissue of the udder, in dairy cows, and presents a huge economic problem for the dairy industry worldwide. Thus, control and treatment of mastitis in dairy cows is vital in order to reduce the costs associated with the disease. The main purpose of the current work was to examine the current dosage of rifaximin for the treatment mastitis in cows caused by *S. aureus* using pharmacokinetic/pharmacodynamic integration in a mouse mastitis model. The mouse mastitis model was established via injection of *S. aureus* Newbould 305 (400 CFU/gland) into the mouse mammary gland. A single dose of 50, 100, 200, or 400 μg/gland, administered via intramammary infusion, was used to study the pharmacokinetics of rifaximin. The pharmacokinetic parameters were analyzed by non-compartment and non-linear mixed-effect models using Phoenix software (version 8.1; Pharsight, USA). *In vivo* pharmacodynamics was used to examine 18 therapeutic regimens covering various doses ranging from 25 to 800 μg/gland and three dosing intervals of 8, 12, and 24 h per 24 h experiment cycle. The antibacterial effect of rifaximin was elevated with higher concentrations of rifaximin or shorter intervals of administration. The percentage of time that drug concentrations exceeded the MIC during a dose interval (%T > MIC) was generally 100% for rifaximin and was not better than AUC_24_/MIC in the sigmoid *E*_max_ model of inhibitory effect. The optimal antibacterial effect was 2log_10_CFU/gland when the magnitude of AUC_24_/MIC reached 14,281.63 h. A total of 14,281.63 h of AUC_24_/MIC was defined as a target value in the Monte Carlo simulation. The clinically recommended dosage regimen of 100 mg/gland every 8 h in 1 day achieved an 82.97% cure rate for the treatment of bovine mastitis caused by *Staphylococcus aureus* infection.

## Introduction

Cow mastitis is an inflammation of the mammary gland that can cause huge economic losses to the dairy cow industry due to the decline in milk quality, treatment costs, and prohibitive labor costs (1–3). Mastitis can be divided into two categories according to clinical characteristics: clinical and subclinical mastitis, both of which are usually caused by bacteria, though some infections are due to fungus, yeast, or even algae (4, 5). *Staphylococcus aureus* is a common Gram-positive bacterium and the main pathogenic bacteria that causes cow mastitis (6). *Staphylococcus aureus* can also invade the epithelial barrier and gain access to internal tissues, and it can infect almost any organ and cause a broad spectrum of diseases including pneumonia, osteomyelitis, sepsis, and endocarditis. Treatment of these infections has been complicated because of its ability to persist in tissues and to evade the host immune response (7, 8). Antibiotics are undoubtedly the best method to treat cow mastitis induced by *S. aureus*. Rifaximin is a derivative of rifamycin and has a broad spectrum of antibacterial activity, against Gram-positive and Gram-negative microorganisms, as well as both aerobes and anaerobes (9). Rifaximin has been approved for the treatment of hepatic encephalopathy, bacterial diarrhea, as well as acute and chronic bacterial infection of the gut in humans (10, 11). Studies have shown that the bioavailability of rifaximin by oral administration is very low and that it is not absorbed by the intestinal mucosa either in healthy individuals or in those with pathological conditions. Therefore, rifaximin can have local effects in the gut after oral administration (12). In veterinary medicine, rifaximin is a good alternative to metronidazole for the treatment of the chronic enteropathy in dogs (13). Moreover, it is also used for the treatment of endometritis and mastitis in dairy cows (14, 15). Rifaximin intramammary infusion (lactating cows or dry cows) has been approved to treat cow mastitis. The currently approved rifaximin intramammary infusion dosage regimen approved for the treatment of bovine *S. aureus* mastitis is 100 mg/gland (16).

Irrational use of antibiotics can lead to the emergence of bacterial resistance; thus, the dosages of antibiotics used in clinical and veterinary medicine should be optimized. Pharmacokinetic and pharmacodynamic (PK/PD) modeling is a particularly good method that can concurrently analyze the time course and the antibacterial effectiveness of a drug. The PK/PD method can also elucidate an inadequate daily dose or extended dosing interval under a traditional dosing regimen and provide a better dose regimen for curing bacterial diseases. Animal experiments are indispensable in PK/PD modeling, though using large animals, such as cattle or sheep for mastitis experiments, is very expensive. The mouse model of mastitis has the potential to greatly assist in the development of new drug treatments prior to further testing in clinical trials, to assess the rationality of dosages of current drugs, and to improve our understanding of the relationships between the microbiome, the host immune response, and lactation (17–19). This study was a post-approval study, which was to analyze the population pharmacokinetics of rifaximin in mammary gland tissues of healthy mice and the pharmacodynamics of rifaximin on mammary gland tissues in mice with mastitis induced by *S. aureus*. The pharmacokinetics and pharmacodynamics of rifaximin were then combined using the inhibitory sigmoid *E*_max_ model to obtain accurate surrogate PK/PD indexes under different levels of antibacterial activity. The PK/PD profiles were extrapolated for the treatment of clinical mastitis in cows, and the clinical dosage regimen was evaluated using the Monte Carlo simulation.

## Materials and Methods

### Bacterial Strains and Animals

*Staphylococcus aureus* Newbould 305 (ATCC 29740), a bacterial strain isolated from a clinical case of cow mastitis in Orangeville, Ontario, Canada, in 1958, was used for the current study. This strain is the standard strain used for inducing experimental intramammary infection (IMI) in cows (20). The strain was provided by Shenzhen Huada Gene Company. In addition, 41 *S. aureus* clinical isolates were collected from dairy farms in Beijing suburbs in 2017–2019. Each of the 41 clinical isolates was isolated from per dairy cow with mastitis separately, which was identified by chromogenic medium, microscope and PCR. These cows had never been treated with rifaximin by the time we collected clinical *S. aureus* isolates. Lactating CD-1 mice, weighing 30–45 g, were purchased from Vital River Laboratories in Beijing China. Mice were raised in a special-pathogen-free (SPF) environment with 1 lactating mouse and 10 suckling mice per cage in a 12:12 light:dark cycle. Mice had free access to food and water. All mouse experiments were approved by the Animal Use and Care Committee of Chinese Academy of Agricultural Sciences, and the guidelines of the American Association for Accreditation of Laboratory Animal Care (AAALAC) were followed during all *in vivo* procedures (21).

### Reagents

Standard Rifaximin was purchased from Sigma Chemical Company (St. Louis, Missouri, USA). The standard stock solution of rifaximin was prepared in DMSO at 8,000 μg/ml and stored at −20°C until use. Working solutions were prepared daily by appropriate dilution of the stock solution with DMSO. HPLC grade methanol, acetonitrile, and ammonium formate were purchased from Thermo Fisher Technology Co., Ltd. (China).

### MIC Test

Determination of microbial susceptibility was based on the Clinical and Laboratory Standards Institute (CLSI) guideline (22). The broth microdilution method was performed to determine the minimal inhibitory concentration (MIC) of rifaximin to *S. aureus*. The MICs of 41 clinical *S. aureus* were all determined, and the MIC_50_ and MIC_90_ values were calculated. MIC_50_ and MIC_90_ indicate inhibition of the growth of a bacterial population by at least 50 and 90%, respectively. The MIC of all bacteria was measured three times: all replicates of a given isolate yielded the same MIC value, which was used for subsequent analysis.

### *In vitro* Time-Killing Curves

The *S. aureus* Newbould 305 was cultured overnight, diluted 10-fold, exposed to five different rifaximin concentrations of 0.5×, 1×, 2×, 4×, and 8× MIC, and grown in a shaker at 37°C and a speed of 200 rpm. The growth control group was not added with rifaximin, and the other treatments were the same as the experimental group. *In vitro* time-killing curves were evaluated in the two initial bacterial inoculum of 10^6^ and 10^7^ CFU/ml. A 100 μL volume of the bacterial solution was diluted using a 10-fold serial dilution protocol in 0, 3, 6, 9, and 24 h, and the diluted bacterial solutions were placed on MH agar plates and the bacteria counted. All the MH agar plates were cultured at 37°C for 22–24 h to allow for bacterial growth prior to counting the bacteria.

### Pharmacokinetics of the Mammary Gland

To determine whether drug concentration in one mammary gland affected drug concentration in the other mammary gland, drug was only administered to one mammary gland, and the drug concentration was determined in both mammary glands. Differences in the drug concentrations were analyzed according to the methods described by Yu et al. (1). The fourth pair of mammary glands in CD-1 mice are the largest and easiest to distinguish and obtain; thus, the fourth pair of mammary glands were chosen for the pharmacokinetics experiment. Approximately 1–2 h prior to the experiments, 8–12 day-old offspring were separated from lactating mice. Three mice were selected at each time point. A total of 30 lactating mice were used in this experiment. The mice were anesthetized with pentobarbital, a small opening was made in the mammary duct, and 100 μL of rifaximin (2,000 μg/ml) was injected into the mammary duct using a 33-gauge blunt needle under anatomic mirror. The mice were euthanized with CO_2_ at the corresponding sampling time point. Both the treated and non-treated glands were sampled at 0.08, 0.17, 0.25, 0.5, 1, 4, 8, 10, 12, or 24 h after rifaximin administration. The drug concentrations in the L4 and R4 abdominal glands were determined by HPLC-UV.

Mammary gland PK was carried out in four mice (*n* = 8 glands) after a single dose of 50, 100, 200, or 400 μg/gland rifaximin was administered via intramammary infusion into both the L4 and R4 glands (each gland was treated individually). Four mice were selected at each sampling time in every single dosing group. A total of 160 mice were used for 4 dosing groups. The R4 and L4 mammary gland samples were sampled at 0.08, 0.17, 0.25, 0.5, 1, 4, 8, 10, 12, or 24 h after drug administration. All mammary gland samples were processed, and the residual solution was detected by HPLC-UV.

### Determination of Rifaximin in Mammary Gland Tissue

First, mammary gland tissues were homogenized. Then, 0.5 g of mammary gland tissue homogenate was weighed in a 10 ml polypropylene centrifuge tube, and a 3 ml volume of acetonitrile was added. The mixture was whirled for 1 min using a vortex meter (IKA, Germany) and centrifuged at 7,104 g for 5 min (Thermo Fisher Scientific, USA). The supernatant was transferred to another polypropylene centrifuge tube, and the remaining residue was extracted again with 3 ml volume of acetonitrile. The two extracted solutions were combined and added to a solid-phase extraction (SPE) cartridge (Oasis HLB 3cc 60 mg, Waters Company, USA). The 3 ml acetonitrile was added in the SPE cartridge to elute, and the eluent was evaporated to dry under a nitrogen stream at 40°C. The residue was reconstituted in 1 ml methanol and filtered through a 0.22 μm nylon syringe filter before analysis by HPLC-UV. The conditions for the HPLC-UV analysis were as follows: separation was obtained with a C18 reverse-phase column (Waters XBridge ShieldRP18 4.6 mm×250 mm, 5.0 μm) designed to withstand 4,000 psi, and injection volume was 20 μl. The mobile phases were methanol, acetonitrile, and pH 7.2 ammonium formate, and the composition ratio was 31.5:31.5:37% (V/V/V). The analyses were conducted at a flow rate of 1.4 ml/min in isocratic elution. The running time for one injection was 10 min.

The limits of detection (LOD) and the limits of quantitation (LOQ) were determined via the extracted free mammary gland spiked with known concentrations of rifaximin. The accuracy and precision were determined via the blank mammary gland spiked with three concentrations (10, 50, or 100 μg/g) in five replicates for consecutive 5 days. The recoveries and relative standard deviations (RSD) were calculated to evaluate the accuracy and precision, respectively. The RSD was calculated by RSD (%) = [SD/M] × 100%, where SD represents the standard deviation, and M represents the mean concentration of replicates.

### Pharmacodynamics Trial

The *S. aureus* Newbould 305 was cultured overnight, diluted appropriately, and injected into the mammary gland after mice were anesthetized with pentobarbital, at a concentration of 400 CFU/gland, through a microinjector under an anatomic microscope. The number of bacteria should have reached about 7log_10_CFU/gland after 9 h of growth *in vivo* (23, 24). A 100 μL volume of rifaximin was injected into the L4 and R4 mammary glands of each mouse through a microinjector under an anatomic microscope. A total of 18 dose groups were included in the experimental study. There were three mice in each dose group. Fifty-four lactating mice were used in this experiment. Before administrating rifaximin, the mice were anesthetized with pentobarbital. The treatment dose ranged from 25 to 800 μg/gland, and the interval of administration was 8, 12, or 24 h. The mice were euthanized with CO_2_ 24 h after treatment, and then the mammary gland tissues of mouse were collected for bacterial count before administration and after 24 h. The mammary gland of mice was homogenized by tissue homogenizer, and the homogenate was diluted in a 10-fold serial protocol to count the bacteria. The mammary glands of mice in the non-treated control group were also collected for bacterial count. The pharmacodynamics of rifaximin was expressed as the decrease in bacteria in mammary gland tissues (Δlog_10_CFU/gland).

### PK/PD Analysis

The pharmacokinetic parameters of mammary gland tissues were analyzed by non-compartment and a non-linear mixed-effect model using Phoenix software (version 8.1; Pharsight, USA). The PK/PD parameters, including AUC_24_/MIC (the ratio of area under the concentration-time curve over 24 h to the MIC), %T > MIC (the percentage of time duration of drug concentration exceeding the MIC during a dose interval), and C_max_/MIC (the ratio of peak concentration divided by the MIC), were used as surrogate markers of antibacterial efficacy. The pharmacokinetic parameters of multi-dose administration were obtained by extrapolating the corresponding single dose. The sigmoid *E*_max_ model of inhibitory effect was used to analyze the antimicrobial effect of rifaximin (25), and was described as:

$$\begin{array}{l} E=E_{\max}-\frac{\left(E_{\max}-E_{0}\right)\times C_{e}^{N}}{EC_{50}^{N}+C_{\text{e}}^{N}} \end{array}$$

*E* is the antibacterial effect, which represents changes in the number of bacteria (log_10_CFU/ gland) in the gland sample after 24 h of treatment compared to the initial colony counts; *E*_max_ is the log_10_CFU/gland in the drug-free control sample; *E*_0_ is the log_10_CFU/gland in the test sample containing rifaximin, when the maximum antibacterial effect was achieved; *C*_*e*_ is the PK/PD index (AUC_24_/MIC, *C*_max_/MIC, %T > MIC); *EC*_50_ is the value of PK/PD index of drug producing 50% of the maximum antibacterial effect; and *N* is the Hill coefficient, which describes the steepness of the concentration-effect curve.

### Monte Carlo Simulation

Three required parameters must be provided for Monte Carlo simulation, using Crystal Ball Professional V7.2.2 software, to perform 10,000 sessions: PK/PD target magnitude, the AUC in cow milk, and the prevalence and distribution of MIC. Based on our team's previous PK study of rifaximin in cows, the AUC of the milk sample was assumed to be lognormal distribution with a mean value and standard deviation of 340.73 ± 43.968 h·μg/ml. The prevalence and distribution of MIC of clinical *S. aureus* being 0.031, 0.063, and 0.125 μg/ml were 0.634, 0.268, and 0.098, respectively. The target values of AUC/MIC for 1.5log_10_CFU/gland or 2log_10_CFU/gland bacterial reduction were obtained in the mouse mastitis model experiment using the sigmoid *E*_max_ model of inhibitory effect. Thus, the efficacy probability of clinical 100 mg/gland of rifaximin by mammary gland injection at different administration intervals could be obtained, and was defined as the probability of target attainment (PTA).

## Results

### Distribution of MICs

The MICs of 41 strains of mastitis bacterial isolates ranged from 0.031 to 0.125 μg/ml, the MIC of the *S. aureus* reference strain 0.0625 μg/ml, and the detailed MIC of each clinical strain is shown in Table 1. The MIC_50_ and MIC_90_ of the 41 strains of mastitis bacterial isolates were calculated as 0.031 and 0.063 μg/ml, respectively.

**Table 1:** The MIC of rifaximin against 41 clinical isolates.

**Name of bacteria**	**MIC (μg/ml)**	**Name of bacteria**	**MIC (μg/ml)**	**Name of bacteria**	**MIC (μg/ml)**
BJK8C049	0.031	BJG7C046	0.031	BJB8C035	0.031
BJK8C055	0.063	BJG7C052	0.063	BJN8C130	0.031
BJK8C060	0.125	BJG7C056	0.125	BJN8C133	0.031
BJK8C063	0.031	BJG7C065	0.031	BJN8C141	0.063
BJK8C018	0.031	BJG7C066	0.031	BJN8C151	0.031
BJN8C012	0.063	BJG7C068	0.031	BJN8C152	0.031
BJN8C018	0.063	BJG7C070	0.031	BJN8C154	0.031
BJN8C021	0.063	BJG7C074	0.125	BJN8C155	0.063
BJN8C036	0.063	BJG7C076	0.031	BJN8C174	0.125
BJN8C041	0.031	BJG7C077	0.031	BJD8C026	0.031
BJN8C054	0.063	BJG7C080	0.031	BJD8C027	0.031
BJG7C039	0.031	BJG7C082	0.031	BJD8C030	0.031
BJG7C040	0.063	BJG7C084	0.031	BJB7C017	0.031
BJB7C018	0.063	BJB7C021	0.031		

### *In vitro* Time Killing Curves of Rifaximin Against Different Inoculum Loads

**Figure 1:**
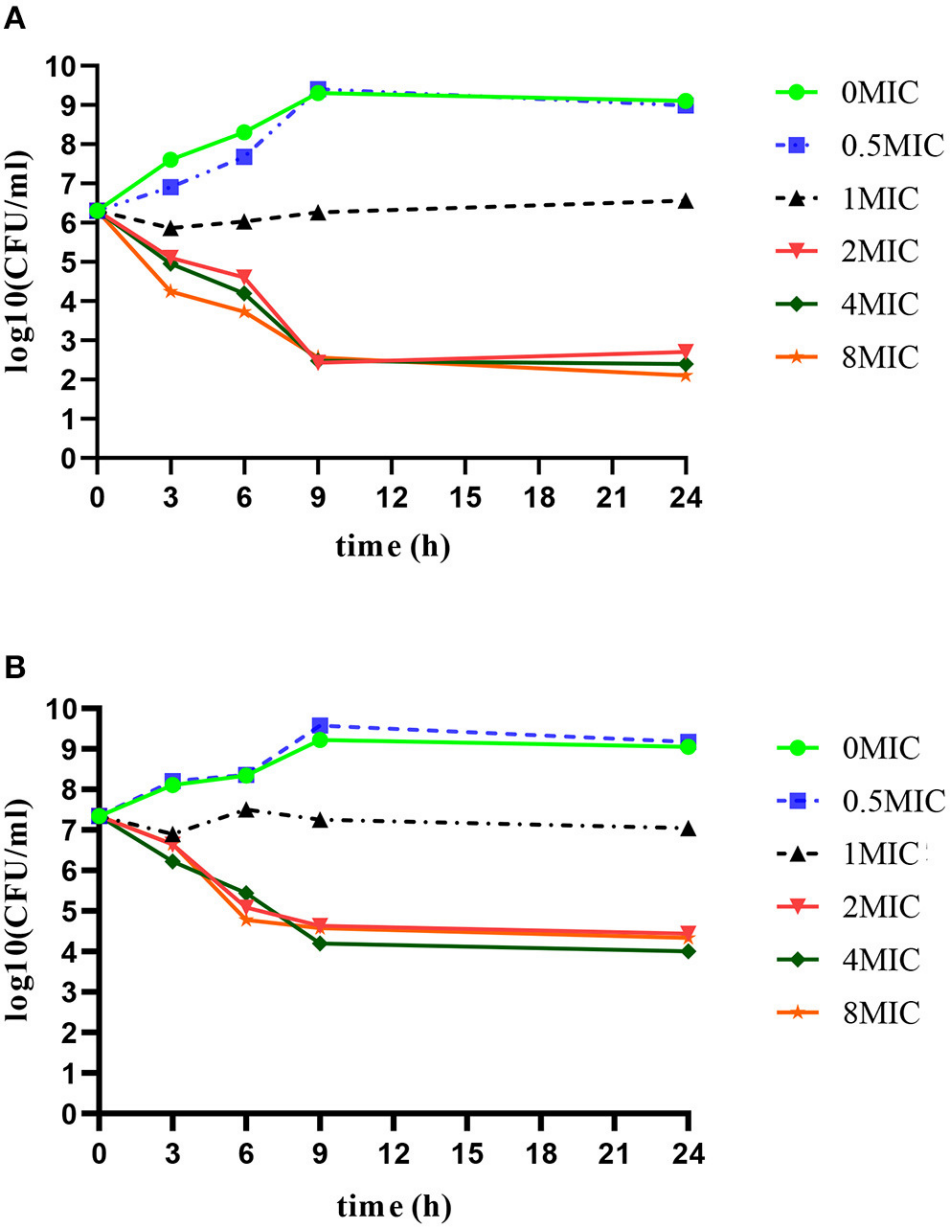
*In vitro* time bactericidal curve of rifaximin against *S. aureus* Newbould305 with different initial bacterial load. **(A)** 106 CFU/ml initial inoculum group. **(B)** 107 CFU/ml initial inoculum group.

Time killing curves of rifaximin *in vitro* were determined in 10^6^ CFU/ml and 10^7^ CFU/ml initial *S. aureus* Newbould 305 inocula and are shown in Figure 1. The bactericidal effect of rifaximin on *S. aureus* showed a time-dependent relationship, as the killing speed did not change, and the killing level was maintained when the concentration of rifaximin reached more than two MIC. Initial *S. aureus* Newbould 305 inocula of 10^6^ CFU/ml and 10^7^ CFU/ml, treated with 2 MIC and higher concentrations of rifaximin, decreased by 4logCFU/ml and 3logCFU/ml, respectively; however, the bacteria continued to grow when the initial concentration of rifaximin was below the MIC. The bacterial killing rate (CFU/h) during the initial 9 h of exposure to rifaximin was calculated. The bacterial killing rate of 10^6^ CFU/ml initial inoculum was almost 2.1 × 10^5^ CFU/h at 2 × MIC, 4 × MIC, and 8 × MIC during the initial 9 h of exposure to rifaximin. The bacterial killing rate of 10^7^ CFU/ml initial inoculum was almost 2.4 × 10^6^ CFU/h at 2 × MIC, 4 × MIC, and 8 × MIC during the initial 9 h of exposure to rifaximin.

### Pharmacokinetics of Rifaximin in Mammary Gland Tissue

LOD and LOQ were 200 and 400 ng/g, respectively, in the mammary glands. The average recoveries for 10, 50, and 100 μg/g were 79.25 ± 4.77, 83.24 ± 3.70, and 85.07 ± 4.87%, respectively. Furthermore, the ranges of intra RSD and inter RSD were 2.24–10.06 and 4.42–6.01%, respectively. Detailed values are shown in Table 2.

**Table 2:** The average recovery, intra RSD and inter RSD of rifaximin in mammary gland at three spiked concentration.

**Spiked concentration (μg/g)**	**Average recovery (%)**					**SD (%)**	**Intra RSD (%)**	**Inter RSD (%)**
			**1 d**	**2 d**	**3 d**	**4 d**	**5 d**	
10	79.25	4.77	7.33	2.24	3.88	3.24	7.61	6.01
50	83.24	3.70	2.91	2.92	4.46	3.00	7.16	4.42
100	85.07	4.87	6.37	5.13	2.56	3.54	10.06	5.73

**Figure 2:**
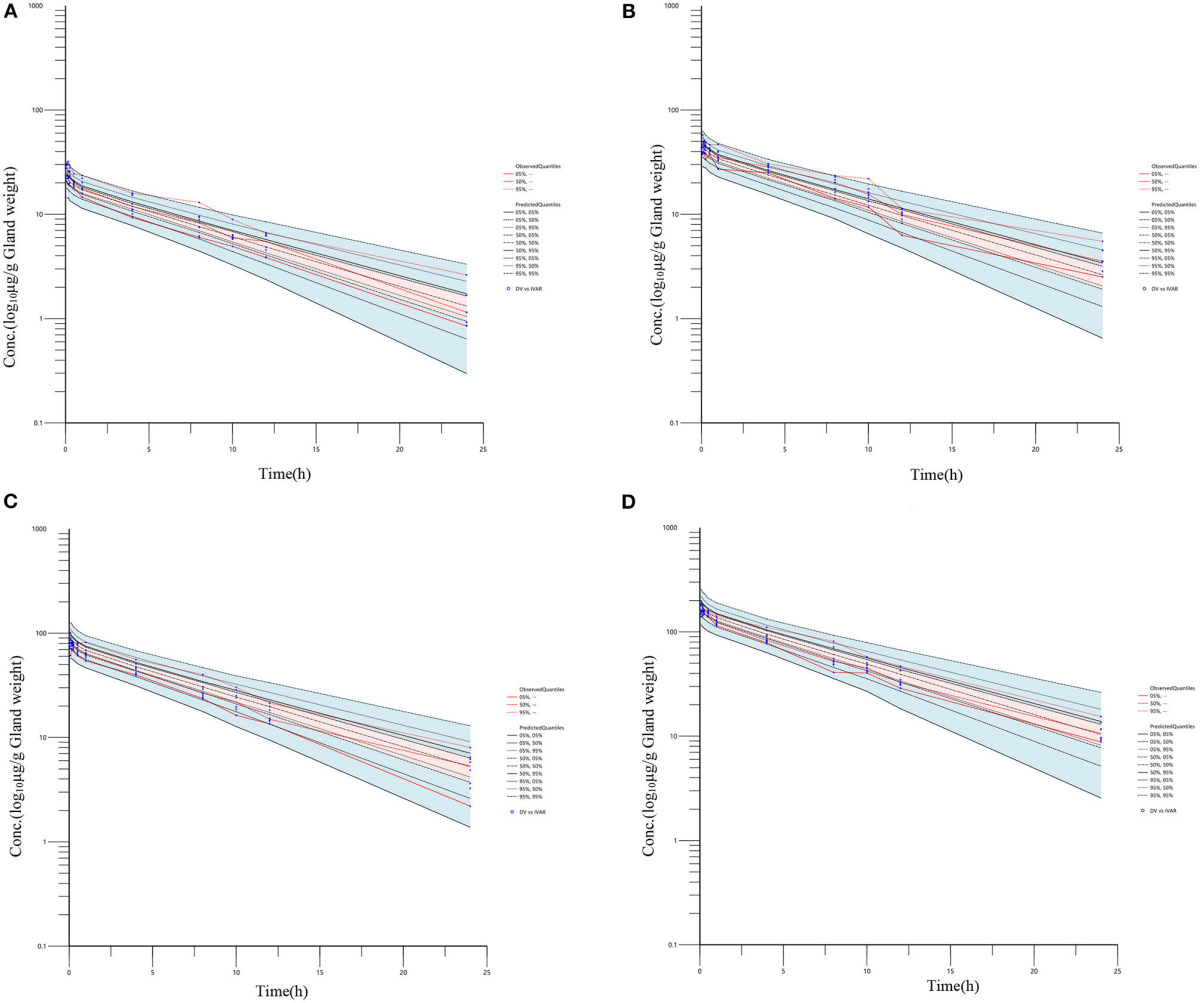
Rifaximin concentration vs. time profile in mammary gland following an intramammary administration dose of 50, 100, 200, and 400 μg/gland. Plotted symbols show the observed concentration data, and the lines show the results of visual predictive checks. For simulated data, the 5th, 50th and 95th percentiles of the final model simulated data are displayed; for real data, the 5th, 50th, and 95th percentiles of the observed concentrations are displayed. (**A–D** represents 50, 100, 200, and 400 μg/gland, respectively).

In the pilot study, the rifaximin concentration of the untreated mammary gland was at or below the lower limit of quantification. Four different single doses of rifaximin (50, 100, 200, or 400 μg/gland), given via intramammary administration, were used to study the pharmacokinetics of rifaximin in mammary gland tissue. The concentration of rifaximin at different time points was analyzed using non-compartment and non-linear mixed-effect models. The visual predictive check (VPC) stratified by dose of rifaximin are displayed in Figure 2. Based on the criteria of the smaller Akaike information criterion (AIC) and the better goodness of fit, the two compartments model was selected as the most suitable model for non-linear mixed-effect model. The pharmacokinetic parameters of rifaximin in mammary gland tissue analyzed by non-compartment and the estimative values of fixed effect parameters analyzed by non-linear mixed-effect model are shown in Tables 3, 4, respectively. Elimination half-life T_1/2_ and the mean residence time (MRT) were 6.38, 6.78 h, respectively, when analyzed by non-compartment. PK parameters of multiple doses of rifaximin were extrapolated from the values obtained in the study described above.

**Table 3:** The main pharmacokinetic parameters of rifaximin in mammary gland following an intramammary administration with single dose of 50, 100, 200, and 400 μg/gland in mice and analyzed by non-compartment model.

**Parameters**	**Administered dose (μg/gland) (*****n*** **=** **8)**				
	**50**	**100**	**200**	**400**	**Mean ± SD**
**Non-compartment model**					
T_1/2_ (h)	5.98	6.60	6.16	6.77	6.38 ± 0.37
MRT (h)	6.66	6.97	6.65	6.84	6.78 ± 0.15
AUC_24_ (h·μg/g)	176.83	373.35	602.01	1,215.86	
*C*_max_ (μg/g)	25.82	45.32	80.52	165.23	

**Table 4:** Population pharmacokinetic parameters of rifaximin in mammary gland using the NONMEM model.

**Parameter**	**Estimate**	**Units**	**SD**	**CV%**
V1	2.15	g	0.07	3.22
V2	0.46	g	0.09	18.84
Cl1	0.29	g/h	0.00	1.57
Cl2	0.89	g/h	0.34	37.77
α	2.38	1/h	0.84	35.39
ß	0.11	1/h	0.00	2.77

*V_1_, Central compartment volume of distribution; V_2_, Peripheral compartment volume of distribution; Cl_1_, Clearance of the central compartment; Cl_2_, Intercompartmental distribution; *α*, Distribution rate constant; *ß*, Elimination rate constant*.

### Pharmacodynamics of Rifaximin With Different Administration Regimens

In the study of construction of mastitis mice model, no death was observed within 9 h. All mice developed slight rough pelage and visible swelling of the mammary gland. The mammary gland showed severe clinical signs, such as bloody exudation and dilated vessels after dissection.

**Figure 3:**
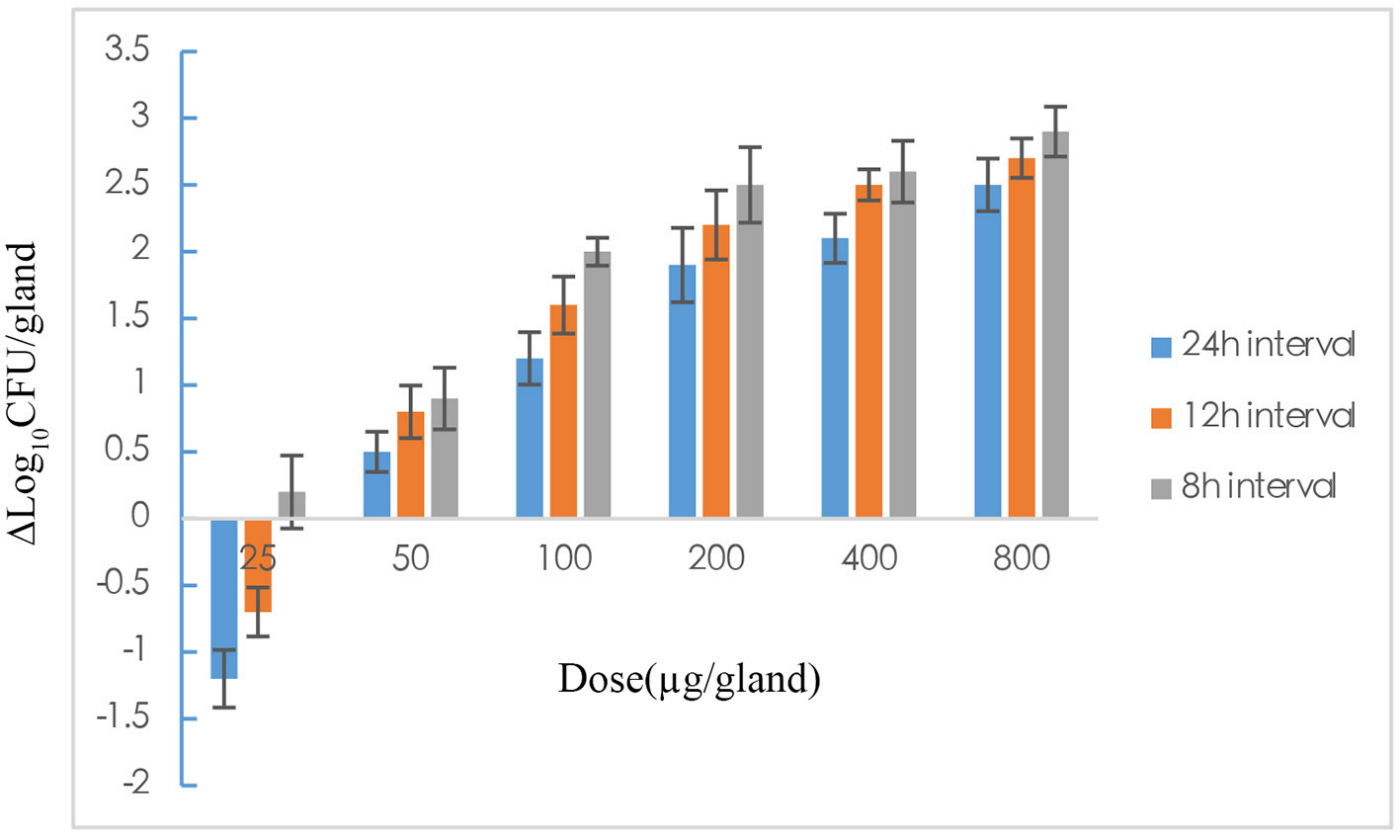
The effect of rifaximin on mastitis induced by *Staphylococcus aureus* in mouse after 18 dose regimens designed with 25, 50, 100, 200, 400, and 800 μg/gland and dosing intervals 8, 12, and 24 h, which was expressed as Δlog_l0_CFU/gland.

The pharmacodynamics of rifaximin with different administration regimens in the mastitis mouse model are expressed by decreases in the bacterial level. The 18 dosing regimens were included 6 doses (25, 50, 100, 200, 400, and 800 μg/gland) and three dosing intervals (8, 12, and 24 h). The pharmacodynamics of different administration regimens are shown in Figure 3. In all single-dose groups, the maximum antibacterial effect, 2.5log_10_CFU/gland reduction, was obtained with the highest dose (800 μg/gland). The pharmacodynamics of rifaximin increased as the dosage increased, and an improvement in pharmacodynamics was also found in the 8 and 12 h dosing interval groups at the end of the experiment. Pharmacodynamics were better in the 8 and 12 h interval 200 μg/gland groups compared with the single-dose 400 and 800 μg/gland groups.

### Integration of PK/PD Parameters

**Figure 4:**
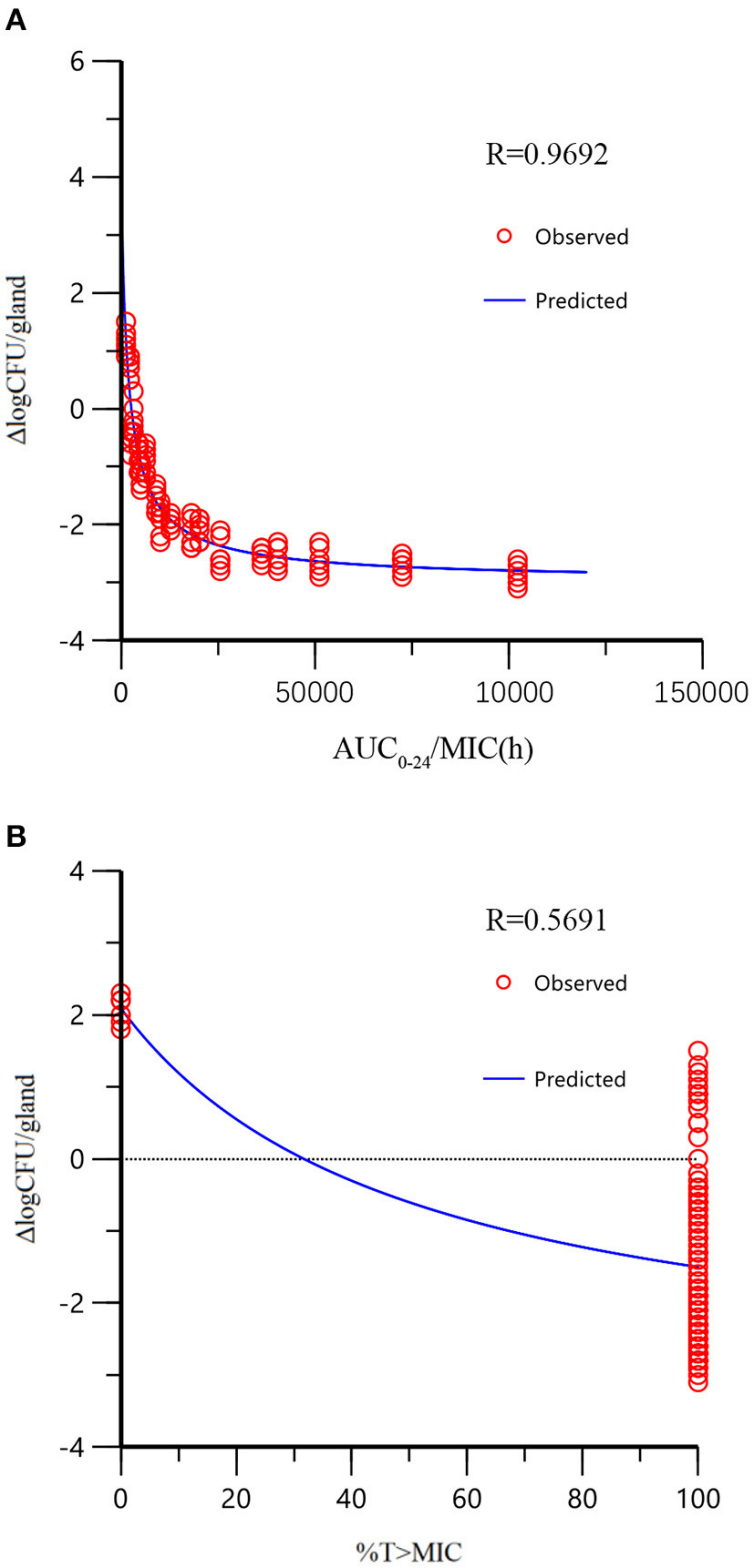
The relationship between PK/PD parameters and bactericidal effect of rifaximin (Δlog_10_CFU/gland) analyzed by the sigmoid model. The dots represented the antibacterial effect of rifaximin (*E* = final log_10_CFU/gland – initial log_l0_CFU/gland) and the line represented the predicted value of *E*. The correlation of %T > MIC with Δlog_10_CFU/gland was very low because of the distribution of %T > MIC (either 100 or 0%), which was not appropriate for PK/PD integration (there are only two independent values, so *R*^2^ has no meaning for %T > MIC).

The 2 alternative PK/PD parameters, including %T > MIC and AUC_24_/MIC, were used in the sigmoid *E*_max_ model of inhibitory effect, and the correlation coefficients (R^2^) were 0.57 and 0.97, respectively. In the mouse mammary glands, the concentration of rifaximin was higher than MIC at all time-points during the experimental period, and the %T > MIC_90_ was 100% during the 24 h experimental cycle. Since the correlation between %T > MIC_90_ and Δlog_10_CFU/gland was very poor in the sigmoid *E*_max_ model of inhibitory (*R*^2^ =0.57), the AUC_24_/MIC_90_ was chosen the best alternative parameter and was selected for evaluating the correlation between PK and PD activity in the mammary gland. As shown in Figure 4A, the tail of the simulation curve leveled off when the Δlog_10_CFU/gland approached the 2log_10_CFU/gland reduction; thus, adding doses was not necessary to achieve a higher antibacterial effect, and the clinical symptoms of redness, swelling, and heat had been significantly improved. Therefore, the 2log_10_CFU/gland reduction was the best in the model and its corresponding AUC/MIC_90_ was used as a target value for Monte Carlo simulation. The important parameters in the AUC24/MIC using the inhibitory form *E*_max_ sigmoid model after intramammary administration was shown in Table 5.

**Table 5:** The important parameters in the AUC_24_/MIC using the inhibitory form *E*_max_ sigmoid model after intramammary administration.

**Parameters**	**AUC_**24**_/MIC**
Log *E*_max_ (log_10_CFU/gland)	4.33 ± 2.00
Log *E*_max_-Log *E*_0_ (log_10_CFU/gland)	7.32 ± 2.14
*EC*_50_ (h)	1,734.33 ± 1,004.34
For1.5log_10_CFU/gland reduction	8,173.48
For2log_10_CFU/gland reduction	14,281.63
Slope (*N*)	0.88 ± 0.18

### Monte Carlo Simulation

**Figure 5:**
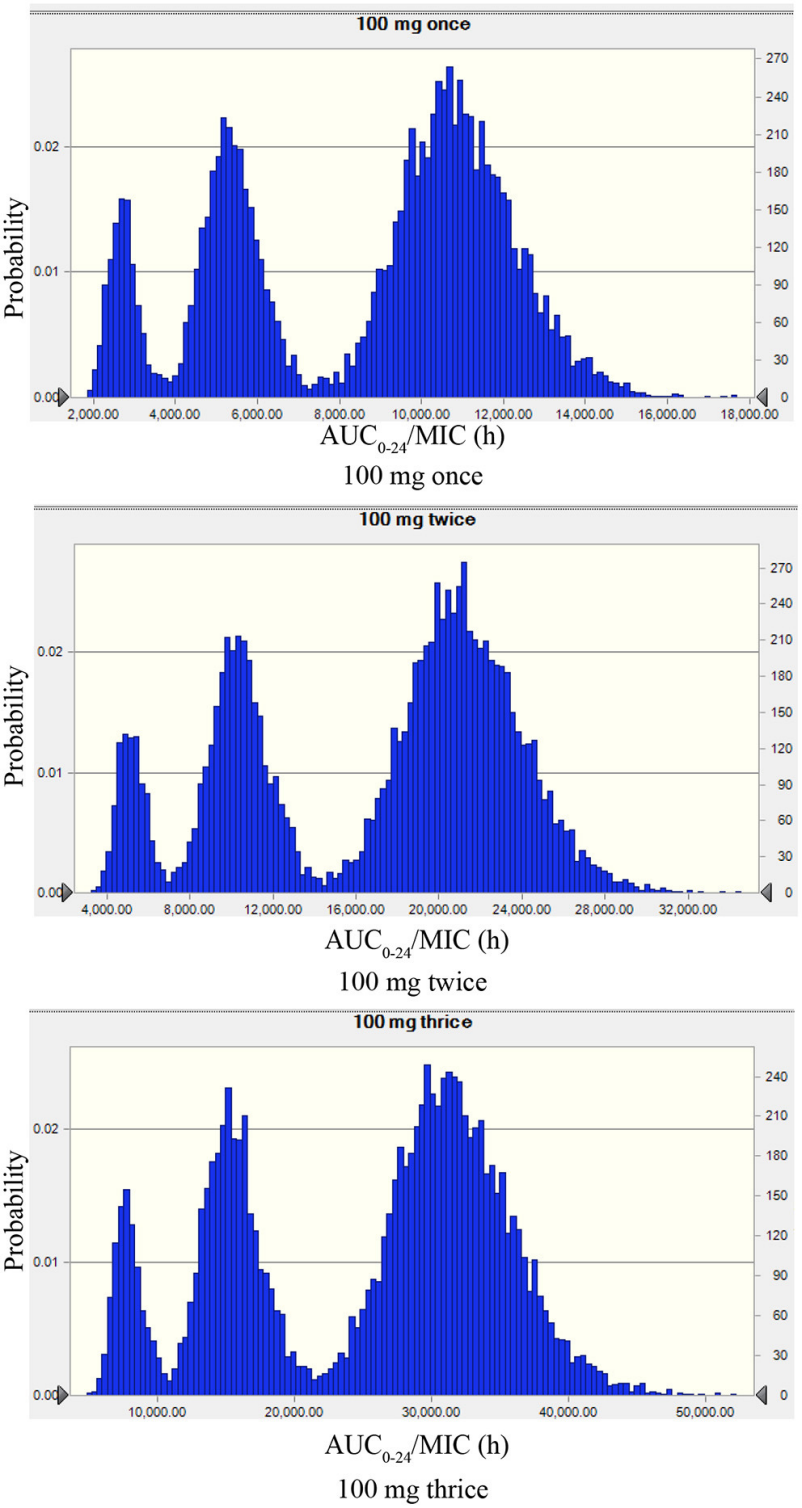
The probability distribution plots of AUC/MIC analyzed by Monte Carlo simulation mimicking rifaximin dosing regimens of 100 mg/gland by intramammary infusion once, twice, and three times. The peak on the left, the middle, and the right represents the AUC/MIC plotted distribution of the highest MIC (0.125 μg/ml), the median MIC (0.063 μg/ml), and the lowest MIC (0.031 μg/ml).

**Figure 6:**
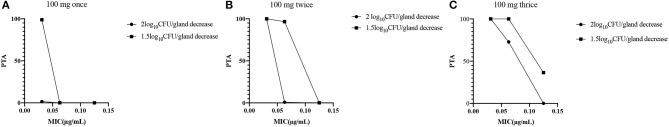
**(A)** Fractional attainment of the 2log_10_CFU/gland decrease for *S. aureus* (•) and 1.5 log_10_CFU/gland decrease for *S. aureus* (■) for the 1-dose (100 mg/gland)/24 h administration. **(B)** Fractional attainment of the 2log_10_CFU/gland decrease for *S. aureus* (•) and 1.5log_10_CFU/gland decrease for *S. aureus* (■) for the 2-dose (100 mg/gland)/24 h administration. **(C)** Fractional attainment of the 2log_10_CFU/gland decrease for *S. aureus* (•) and 1.5log_10_CFU/gland decrease for *S. aureus* (■) for the 3-dose (100 mg/gland)/24 h administration.

The recommended dose of rifaximin for the treatment of cow mastitis is 100 mg/gland (16). Three administration regimens were chosen for the Monte Carlo simulation: one dose was given every 8, 12, or 24 h so that the total doses in the experimental period were 3, 2, and 1, respectively. The probability distribution plots of AUC/MIC analyzed by Monte Carlo simulation was shown in Figure 5. When the target value of AUC_24_/MIC was set for a 1.5log_10_CFU/gland decrease, the calculated PTA for the 3-dose/24 h administration was 93.77%, and the PTA for the 2log_10_CFU/gland decrease was 82.97%. The detailed values of PTAs for different treatment schemes are shown in Table 6 and Figure 6.

**Table 6:** The PTA of AUC/MIC for 1.5 or 2log_10_CFU/gland decrease after 100 mg/gland once, twice, and thrice by intramammary administration in cows (PTA was defined as the probability of target attainment that the efficacy probability of clinical 100 mg/gland of rifaximin was obtained by mammary gland injection at different administration intervals).

**Dose regimen**	**PTA** ** (1.5log_**10**_CFU/gland decrease)**	**PTA** ** (2log10CFU/gland decrease)**
100 mg once	62.80%	1.04%
100 mg twice	89.06%	64.11%
100 mg thrice	93.77%	82.97%

## Discussion

Mastitis is an inflammatory response resulting from an infection of udder tissue and has been reported in numerous species, including cows, goats, and sheep (26–28). It is very difficult to use cows and goats for studies of mastitis due to problems with management and high costs. Mouse is a typical pattern animal, which is cheap and easy to obtain. Like the dairy cow, it also possesses one teat duct or streak canal per teat. Each mammary gland of the mouse is anatomically and functionally distinct from its neighbor in the same manner as in the dairy cow (29). Many literature reports have confirmed that the pathological phenomena of mammary tissues of mice infected by bacteria are very similar to that of cow mastitis, such as neutrophil infiltration, tissue damage, etc. (24). The results obtained from the mouse mastitis model can provide basis and inspiration for the treatment of cow mastitis. Therefore, mice models are often used to study mastitis. In 1970, Chandler et al. (29) first developed a mouse model of mastitis induced by bacteria, including *S. aureus, Streptococcus agalactiae, Corynebacterium pyogenes, Escherichia coli*, and *Pseudomonas aeruginosa*. The *S. aureus* mastitis mouse model was then successfully established by many researchers and has been used to study the pathogenesis of cow mastitis and the anti-inflammatory signal pathway of some natural active ingredients such as Salidroside and Allicin (30–32). The mouse model was also used to define drugs disposition and PK profile. Yu studied the pharmacokinetics of cefquinome in the mammary gland of mice to simulate its pharmacokinetics in dairy cows (1, 25). In this study, 400 CFU/gland of *S. aureus* Newbould 305 were injected into one mammary gland of each mouse. After 9 h of growth *in vivo*, the number of bacteria had reached about 7log_10_CFU/gland, which was consistent with results in a study by Yu et al. (23). The feed intake, water intake, and activity of the mice infected with bacteria decreased significantly. Mammary gland redness and swelling were noted with some glands feeling very hard. In addition, the milk from some of the mammary glands had a foul odor. These clinical symptoms were consistent with previous reports (33–35). These characteristics further confirmed the successful establishment of a mouse mastitis model that could be used in the subsequent rifaximin pharmacodynamic studies.

In the MIC study, the MIC of rifaximin against *S. aureus* ranged from 0.031 to 0.125 μg/ml, but was mainly distributed at 0.031 mg/ml, which was similar to results reported in previous papers (36, 37). The MIC_50_ and MIC_90_ were calculated with 0.031 and 0.063 μg/ml, respectively, and the MIC_90_ of 0.063 μg/ml was used in the PK/PD model. The *in vitro* time-killing curves indicated that the 2MIC and higher concentrations had bactericidal effects as shown by the 4log_10_CFU/ml reduction in the 10^6^ CFU/ml initial inoculum group and the 3log_10_CFU/ml reduction in the 10^7^ CFU/ml initial inoculum group. A higher initial bacteria inoculum may require a higher concentration of antibiotic or a longer exposure time to the antibiotic. Buldain made a time-kill curve for the effect of rifaximin on *S. aureus* ATCC 29213 at pH 5.0. They found an almost 4log_10_CFU/ml reduction in the 10^6^ CFU/ml initial inoculum group with 2MIC or higher concentrations of rifaximin; however, rifaximin did not inhibit the growth of bacteria when the concentration was below the MIC (36). These results are consistent with those of the current study, showing that the effect of rifaximin is time dependent. In the *in vivo* pharmacodynamics experiment in this study, the efficacy of rifaximin increased with the increase in dosage, and the shortened administration interval, and the maximum antibacterial effect of 2.9log_10_CFU/gland was found with a dosage of 800 μg/gland given at an 8 h dosing interval. Yu et al. (1) studied the pharmacodynamics of cefquinome on mouse mastitis induced by *S. aureus* Newbould 305 and found a maximum antibacterial effect of almost 2log_10_CFU/gland with 800 μg/gland and an 8 h dosing interval.

In the pilot study of rifaximin in mammary gland, the concentration of rifaximin of the untreated mammary gland homogenate was lower than or equal to the limit of quantitation, which is consistent with the blood-milk barrier (4). The blood-milk barrier is formed by mammary gland secretory cells that are closely linked together at their apex by tight junctions, which helps prevent the selective diffusion of drugs between the blood and mammary gland (38, 39). Therefore, the PK parameters in the mammary gland were used for the PK/PD model and Monte Carlo simulation to evaluate and develop a rational regimen for clinical administration of rifaximin.

The results of the pharmacokinetics study in the mouse model showed the elimination half-life (T_1/2_) and mean retention time (MRT) were 6.38 and 6.78 h for the non-compartment model, indicating that rifaximin can reach a high concentration that is maintained for a long time in the mammary glands of mice, while the T_1/2_ and MRT were 5.57 and 7.39 h in the cow milk as analyzed by non-compartment model. By comparing T_1/2_ and MRT in the mice and cow milk, it concludes the disposition of rifaximin PK data in the mouse mammary gland is similar to rifaximin in milk though there are some anatomical differences between mice and cows. Since dairy cows are so important economically, the withdrawal period for milk after rifaximin should be as short as possible. Depletion experiments were also carried out in dairy cows after rifaximin mammary gland injection, and the results showed that the withdrawal period for milk was 95.1 h, which is in accordance with 96 h regulation of the European Veterinary Drug Commission (16). Our pharmacokinetic data in milk have not been published yet, and will be published in another paper.

As shown in Figure 3, the antibacterial effect of rifaximin increased slowly when the dosage was over 200 μg/gland, indicating that the 400 and 800 μg/gland dose regimens were much higher than required to treat mastitis in the mouse model. In Figure 4A, when the Δlog_10_CFU/ml reduction reached two, the antibacterial effect of rifaximin was at a stable level even when the AUC_24_/MIC was increasing, and the flat tail of the curve appeared. A more reasonable dosage, one that occurs before the flat tail of the curve appears, should be selected. When the Δlog_10_CFU/ml reduction reached two, the knee point of the curve appeared (Figure 4), and the corresponding AUC_24_/MIC value was 14,281.63 h. In the sigmoid *E*_max_ model of inhibitory effect, 1.5log_10_CFU/gland was selected as an antibacterial effect value, and the calculated AUC_24_/MIC value was 8,173.48 h. The important parameters in the AUC_24_/MIC using the inhibitory form *E*_max_ sigmoid model after intramammary administration are shown in Table 4.

After dividing this value of 14,282 h by 24 h, we get a scalar (595). It means an average local concentration for the 2log10CFU/gland is 595 times higher than the MIC of the pathogen in a MHB medium. The reasons for this result are as follows. First, unlike other drugs, rifaximin is administered through intramammary administration instead of the traditional way including intravascular or intramuscular. The drug concentration was measured in the mammary gland tissue, which was much higher than that in blood. Second, the total tissue concentrations largely overestimate the free concentrations. Likely, rifaximin is not dispersed evenly in mammary gland tissue, which may lead to a lower concentration of bacteria exposure. The two reasons accounted for the average local concentration 595 times higher than the MIC of the pathogen to get a maximal efficacy. In the next experiment, we will confirm these two reasons.

In the Monte Carlo simulation, 8,173.48 and 14,281.63 h were used for target value decreases of 1.5log_10_CFU/gland and 2log_10_CFU/gland, respectively. The rifaximin PTAs for 100 mg/gland administered once, twice, and three times in 24 h were 62.80, 89.06, and 93.77%, respectively, for a 1.5log_10_CFU/gland decline, and 1.04, 64.11, and 82.97%, respectively, for a 2log_10_CFU/gland decline. The PTAs for three doses in 24 h for the decreases of 1.5log_10_CFU/gland and 2log_10_CFU/gland were all higher than for one or two doses in 24 h. Furthermore, the PTAs of three doses in 24 h for decreases of 1.5log_10_CFU/gland and 2log_10_CFU/gland were all higher than 80%, which is a high cure rate for mastitis. This suggests that a dose of 100 mg/gland rifaximin three times a day via mammary gland injection is reasonable and has a significant antibacterial effect.

As the difference of animal species for cows and mice, we can't copy the results of this study of mastitis mice directly to dairy cows with mastitis. But, the magnitude of PK/PD parameters in mice can still be capable of defining the PK/PD index required for different efficacy outcomes for mastitis cow since various animal infection models including human should share a relatively similar magnitude of the PK/PD index (40).

## Conclusion

Based on the *in vitro* bactericidal curve, rifaximin conformed to a time-dependent antibiotic. When the concentration of rifaximin reached 2MIC, the antibacterial effect did not change even when the concentration increased further; however, the antibacterial effect did increase as the contact time with antibiotics increased. To our knowledge, this is the first study using the PK/PD model to assess the antibacterial effect of rifaximin in a mouse model of mastitis induced by *S. aureus* Newbould 305. The PK/PD model showed that the 1.5log_10_CFU/gland and 2log_10_CFU/gland decreases corresponded to AUC_24_/MICs of 8,173.48 and 14,281.63 h, respectively. The 100 mg/gland clinical administration regimen of rifaximin was evaluated by Monte Carlo simulation, and the results showed 100 mg/gland rifaximin, administered three times a day, resulted in 93.77 and 82.97% cure rates for decreases of 1.5log_10_CFU/gland and 2log_10_CFU/gland in cows with mastitis.

## Data Availability Statement

The original contributions presented in the study are included in the article/supplementary material, further inquiries can be directed to the corresponding author/s.

## Ethics Statement

The animal study was reviewed and approved by the Animal Use and Care Committee of Chinese Academy of Agricultural Sciences.

## Author Contributions

YL and XL proposed the initial experimental conjecture and experimental feasibility analysis. XC provided the technical guidance for bacterial experiment. HW and CC co-completed the experimental technical route writing and finished the whole experiment work together. JZ participated in the construction of mouse mastitis model. HW and CC completed the analysis of experimental data together. HW wrote the final manuscript. All authors approved the final article.

## Conflict of Interest

The authors declare that the research was conducted in the absence of any commercial or financial relationships that could be construed as a potential conflict of interest.
